# Impact of treatment planning using a structure block function on the target and organ doses related to patient movement in cervical esophageal cancer: A phantom study

**DOI:** 10.1002/acm2.12582

**Published:** 2019-04-17

**Authors:** Hidetoshi Shimizu, Koji Sasaki, Makoto Ito, Takahiro Aoyama, Hiroyuki Tachibana, Natsuo Tomita, Chiyoko Makita, Hiroshi Tanaka, Yutaro Koide, Tohru Iwata, Takeshi Kodaira

**Affiliations:** ^1^ Department of Radiation Oncology Aichi Cancer Center Hospital Nagoya Aichi Japan; ^2^ Graduate School of Radiological Technology Gunma Prefectural College of Health Sciences Maebashi Gunma Japan; ^3^ Department of Radiology Aichi Medical University Hospital Nagakute Aichi Japan; ^4^ Department of Radiology Nagoya City University Hospital Nagoya Aichi Japan; ^5^ Department of Radiation Oncology Gifu Prefectural General Medical Center Gifu City Gifu Japan

**Keywords:** directional block, esophageal cancer, helical tomotherapy, patient movement

## Abstract

Helical tomotherapy (HT) can restrict beamlets passing through the virtual contour on computed tomography (CT) image in dose optimization, reducing the dose to organs at risk (OARs). Beamlet restriction limits the incident beamlet angles; thus, the proper planning target volume (PTV) margin may differ from that of the standard treatment plan without beamlet restriction, depending on the patient's movement during dose delivery. Dose distribution changes resulting from patient movement have not been described for treatment plans with beamlet restriction. This study quantified changes in dose distribution to the target and OARs when beamlet restriction is applied to cervical esophageal cancer treatment plan using HT by systematically shifting a phantom. Treatment plans for cervical esophageal cancers with and without beamlet restriction modes [directional block (DB) and nonblock (NB), respectively] were designed for CT images of the RANDO phantom. The PTV margin for the DB mode was set to be the same as that for the NB mode (5 mm). The CT image was intentionally shifted by ±1, ±2, and ±3 voxels in the left–right, anterior–posterior, and superior–inferior directions, and the dose distribution was recalculated for each position using the fluence for the NB or DB mode. When the phantom shift was within the same PTV margin as the NB mode, changes in doses to the targets, lungs, heart, and spinal cord in the DB mode were small as those in the NB mode. In conclusion, the virtual contour shape used in this study would provide safe delivery even with patient movement within the same PTV margin as for the NB mode.

## 1 INTRODUCTION

Helical tomotherapy (HT) is a dose delivery technique, in which the treatment couch moves in the direction of the gantry rotation axis, and the high‐speed driving of 64 multileaf collimators allow the fluence to be finely modulated.^1^ Studies have shown that HT and volume‐modulated arc radiotherapy help improve the dose concentration at the target for cervical esophageal cancers.^2^, ^3^, ^4^, ^5^ However, their use may increase the risk of radiation pneumonitis and pulmonary complications compared to using three‐dimensional (3D) conformal radiation therapy or intensity modulated radiotherapy, because of the increased low‐dose area in the lung.^6^, ^7^, ^8^ Nomura et al^6^ reported that the volumes receiving at least 5, 10, 15, and 20 Gy (V_5Gy_, V_10Gy_, V_15Gy_, and V_20Gy_, respectively) and mean lung dose were significantly associated with the development of symptomatic radiation pneumonitis. Lee et al^7^ observed significant postoperative pulmonary complications after preoperative chemoradiation for esophageal cancer when the V_10Gy_ of the lung was >40%. To address this issue, Chang et al^9^ reported that the pulmonary dose could be reduced in dose optimization of HT using a structure block function, which restricts beamlets that pass through the virtual contour on the computed tomography (CT) image. They used fan‐shaped virtual contours in the lungs, assessed with a virtual esophageal tumor delineated on the CT image of a phantom.^9^ Building on that study, our research group evaluated dose reductions to the organs at risk (OARs) and dose concentrations at the target using various virtual contour shapes for cervical esophageal cancers in 20 patients.^10^ This showed that a semicircular contour following the shape of the lung at a distance of 8 cm from the tracheal bifurcation was the most clinically useful, when the dose reduction to the OARs and the concentration of the dose at the target were considered as a single index.^10^ The restriction of beamlets in dose optimization of HT has been applied to reduce the dose to OARs in various other conditions as well as esophageal cancers.^11^, ^12^, ^13^, ^14^, ^15^ Wojcieszynski et al^11^ reduced doses to the lungs and heart by restricting the beamlets passing through those organs in a simultaneous integrated boost technique for breast cancer. Lee et al^15^ reduced doses to the right hepatic lobe by restricting the beamlets passing through it for locally advanced hepatocellular carcinoma on the left hepatic lobe.

However, beamlet restriction limits the incident beamlet angles; hence, the doses to the targets and OARs may change significantly depending on patient movement during the dose delivery. It has not yet been established whether the planning target volume (PTV) margin in the standard treatment plan without beamlet restriction (e.g., 5 mm) can be applied to the beamlet restriction plan; using the same PTV margin may result in an insufficient dose to the target. In addition, there have been no reports for treatment plans with beamlet restriction related to changes in the dose distribution resulting from patient movement. The aim of this study was to quantify changes in the dose distribution of the target and OARs when beamlet restriction is applied to a cervical esophageal cancer treatment plan using HT by systematically shifting a phantom. For comparison, a standard treatment plan without beamlet restriction was designed, and the difference in the dose distribution change between the treatment plans with and without beamlet restriction was evaluated.

## 2 MATERIALS AND METHODS

### 2.A CT scan and contour delineation

**Figure 1:**
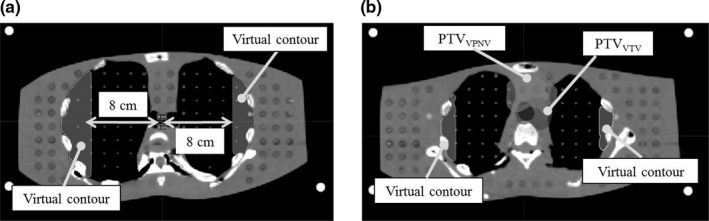
(a) Shape of the virtual contour. This was defined as a semicircle shape following the shape of the lungs at a distance of 8 cm from the tracheal bifurcation. (b) The arrangement of the virtual contour and planning target volumes.

Computed tomography (CT) scans of a RANDO phantom (The Phantom Laboratory, Salem, NY, USA) were acquired to design the treatment plan for cervical esophageal cancer. The scan range was from the supraorbital margin to the inferior margin of the lungs, and the slice thickness and pixel size were 2 and 1.07 mm, respectively. The scanned CT image set was imported into the MIM Maestro software (MIM Software Inc., OH, USA), and the virtual target volume (VTV), virtual prophylactic node volume (VPNV), bilateral lungs, thyroid, heart, and spinal cord were delineated. The PTVs for the VTV and VPNV (PTV_VTV_ and PTV_VPNV_, respectively) were defined by adding isotropic margins of 5 mm. The planning at risk volume (PRV) margin was set at 5 mm for the spinal cord. These margins are those used in standard treatment plans without beamlet restriction. In addition, a semicircular virtual contour following the shape of the lung was drawn at a distance of 8 cm from the tracheal bifurcation to restrict the incident beamlet in the dose optimization process [Fig. 1(a)]. Figure 1(b) shows the positional relationship between the PTVs and the virtual contours. To perform the dose optimization, the CT image set and all the contours were imported into a Tomotherapy Planning Station™ (Accuray Inc., Sunnyvale, CA, USA). The pixel size of the CT images was converted from 1.07 to 2.1 mm based on the Planning Station specification.

### 2.B Dose optimization

**Figure 2:**
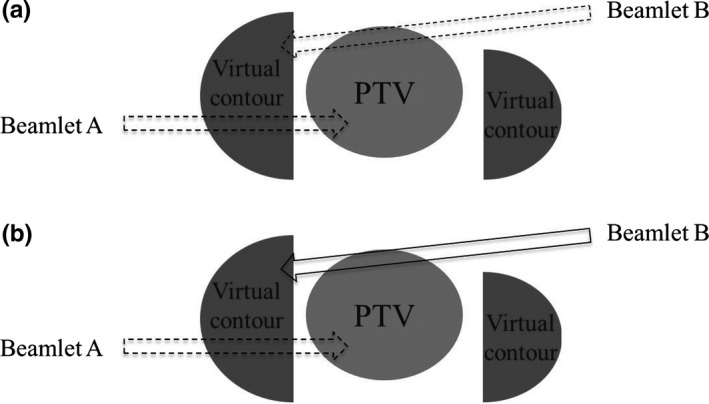
Schematic illustrations of (a) the complete block (CB) mode and (b) the directional block (DB) mode. The CB mode does not use beamlets A and B, which passed through the virtual contour, in the dose optimization process. The DB mode includes beamlet B, which reached the virtual contour after passing through the planning target volume (PTV), in the dose optimization, but it does not include beamlet A, which reached the virtual contour before passing through the PTV.

The Planning Station has a function that restricts beamlets passing through the virtual contour. This function has two modes: complete block (CB) and directional block (DB). Figure 2(a) illustrates the CB mode, showing how beamlets A and B passing through the virtual contour are not included in the dose optimization process. Figure 2(b) illustrates the DB mode, showing how beamlet A, which reached the virtual contour before passing through the PTV, is not included in the dose optimization process, whereas beamlet B, which reached the contour after passing through the PTV, is included. In our previous study, the DB mode with the virtual contour shape used in the present study could create treatment plans for 20 patients without any clinical problems.^10^ We therefore used the DB mode in the present study.

Jaw size, modulation factor, and pitch, which are the treatment planning parameters^16^ of TomoTherapy were set at 2.5 cm, 2.1, and 0.43, respectively . The prescribed dose was set as 60 Gy to the 95% volume of PTV_VTV_ and 48 Gy to the 95% volume of PTV_VPNV_ using the simultaneous integrated boost technique, and it was delivered in 30 fractions. Dose optimization was applied to satisfy the following constraints for PTVs: dose to 98% of the volume (D_98%_) ˃ 54 Gy, D_95%_ ˃ 58.8 Gy, D_50%_ ˂ 64.2 Gy, and D_2%_ ˂ 72 Gy for PTV_VTV_; and D_98%_ ˃ 43.8 Gy, D_95%_ ˃ 46.8 Gy, D_50%_ ˂ 55.8 Gy, and D_2%_ ˂ 64.2 Gy for PTV_VPNV_. Constraints for the OARs were as follows: maximum dose (D_max_) ˂ 130% inside the phantom; D_max_ ˂ 52 Gy and dose to a volume of 1 cm^3^ (D1cm^3^) ˂ 50 Gy for the PRV of the spinal cord; V_10Gy_ ˂ 50%, V_15Gy_ ˂ 40%, and V_20Gy_ ˂ 25% for the lungs. The doses to the thyroid and heart were optimized to be as low as possible.

For comparison, a standard treatment plan without beamlet restriction [nonblock (NB) mode] was designed with the same dose descriptions and dose constraints.

### 2.C Evaluation of the changes in dose with phantom shifts

The CT image set of the phantom used in the above treatment planning was registered in the Planning Station as a verification phantom. The CT image was then shifted ±1, ±2, and ±3 voxels in the left–right (LR), anterior–posterior (AP), and superior–inferior (SI) directions in the DQA Station™ program (Accuray Inc., Sunnyvale, CA, USA) on the Planning Station. The sizes of 1 voxel for the LR, AP, and SI directions were 2.1, 2.1, and 2.0 mm, respectively, and the positive directions for the shift were considered to be right, superior, and anterior. The dose distribution was recalculated for each shifted image using the fluence of the NB or DB mode, and the recalculated dose distribution data were imported into MIM Maestro. The changes in dose distribution with the shifts were evaluated by creating difference images subtracting the recalculated dose distribution with a shift of −1 voxel from that with a shift of +1 voxel, corresponding to a displacement of 4.0 or 4.2 mm. In addition, the dose changes with the phantom shift were evaluated by calculating dose parameters such as D_98%_, average dose (D_mean_), and D1cm^3^ for the VTV, VPNV, heart, spinal cord, thyroid, and each of the right and left lungs.

## 3 RESULTS

### 3.A Comparison of the treatment plans in the NB and DB modes

**Figure 3:**
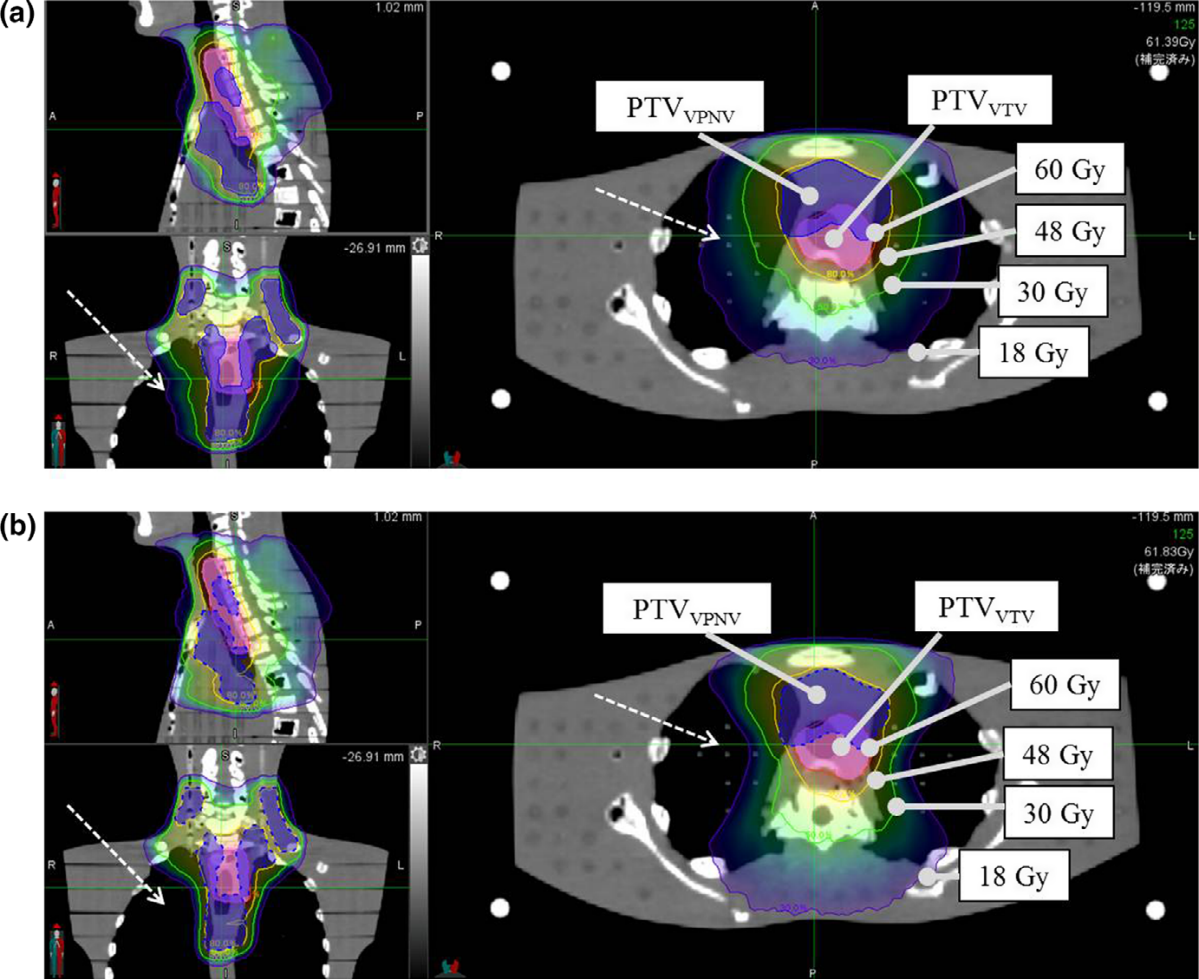
Comparison of the dose distributions with the different planning modes. (a) Nonblock (NB) mode. (b) Directional block (DB) mode. The DB mode reduced the pulmonary dose to a greater extent than the NB mode (dashed arrows).

Figures 3(a) and 3(b) show the treatment plans for the NB and DB modes, respectively. The dose line shape of less than 30 Gy in the DB mode differed from that in the NB mode. Applying the DB mode reduced the low‐dose areas of the lungs, as shown by the dashed arrow in Fig. 3.

### 3.B Dose distribution change resulting from the phantom shift

**Figure 4:**
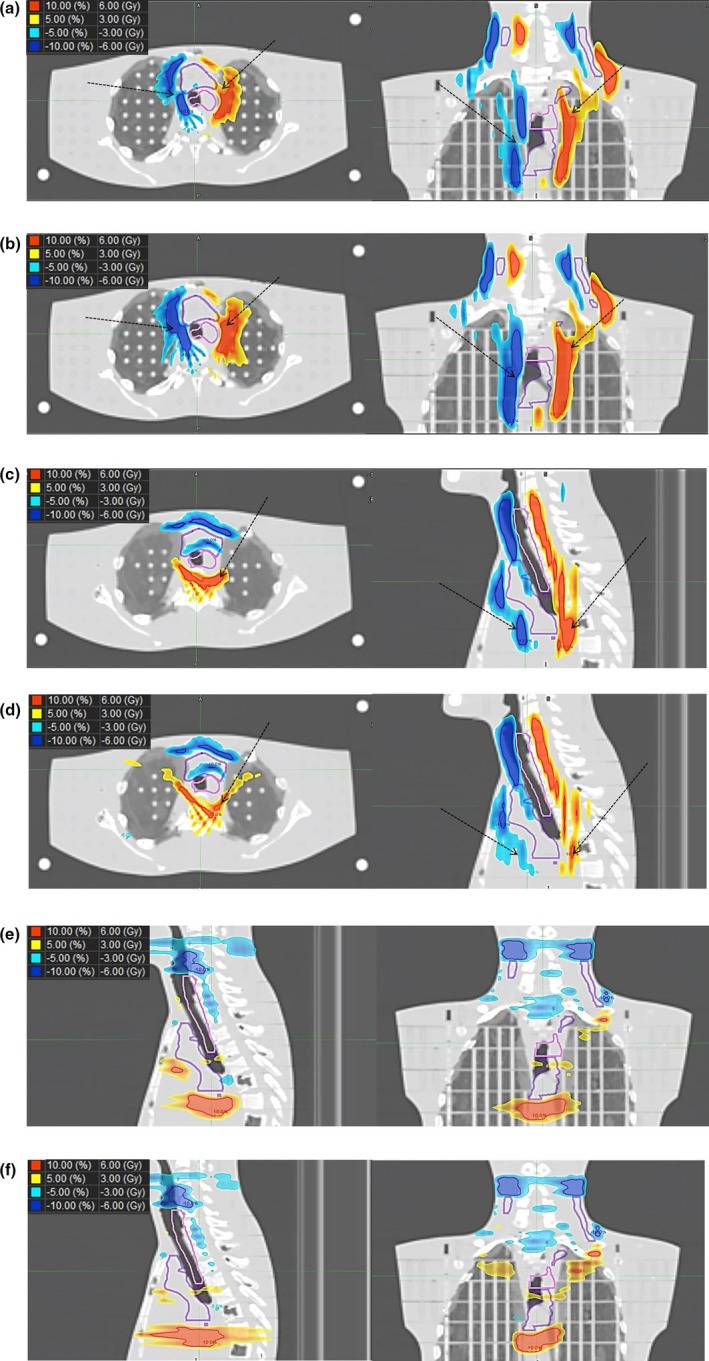
Difference images obtained by subtracting the recalculated dose distribution shifted by −1 voxel from that shifted by +1 voxel. (a) LR direction in the nonblock (NB) mode. (b) LR direction in the directional block (DB) mode. (c) AP direction in the NB mode. (d) AP direction in the DB mode. (e) SI direction in the NB mode. (f) SI direction in the DB mode. The pink and violet contours show the virtual target volume and virtual prophylactic node volume, respectively. AP, anterior–inferior; LR, left–right; SI, superior–inferior.

Figure 4 shows the difference image obtained by subtracting the recalculated dose distribution shifted by −1 voxel from that shifted by +1 voxel. The dose distribution changes in the VTV and VPNV regions (the pink and violet contours, respectively) resulting from the shift in LR direction were greater in the DB mode than in the NB mode [Figs. 4(a), 4(b); dashed arrows]. Conversely, those resulting from the shift in the AP direction were smaller in the DB mode than in the NB mode [Figs. 4(c), 4(d), dashed arrows]. The dose distributions changes did not differ significantly between the NB and DB modes with the shift in the SI direction [Figs. 4(e) and 4(f)]. Table 1 shows the volumes for which the dose distribution difference in Fig. 4 was greater than 6 Gy (equivalent to 10% of the prescribed dose to the PTV_VTV_) or <−6 Gy. The dose distribution changes in the DB mode were quantitatively larger than those in the NB mode for the shift in the LR direction but smaller for the shift in the AP direction.

**Table 1:** Volumes of the dose distribution difference >6 Gy and <−6 Gy in Fig. 4 (cm^3^)

	LR		AP		SI	
	NB	DB	NB	DB	NB	DB
≥6 Gy	55.9	76.7	66.3	43.5	50.3	57.1
≤−6 Gy	62.8	87.0	53.4	42.6	72.4	73.1

AP, anterior–posterior; LR, left–right; SI, superior–inferior; NB, nonblock; DB, directional block; PTV_VTV_, planning target volume for the virtual target volume.

### 3.C Changes in the dose parameters resulting from the phantom shift

Tables S1 and S2 show the dose parameters for the VTV, VPNV, heart, spinal cord, and thyroid in the NB and DB modes. In both modes, the change in dose parameters was small. The dose constraints for PTV_VTV_, PTV_VPNV_, and PRV of the spinal cord in the treatment planning were applied to the VTV, VPNV, and spinal cord, respectively, and when the one‐dimensional (1D) phantom shifts were within two voxels, the dose parameters in the two modes were within the dose constraints. The values in brackets in Tables S1 and S2 indicate the percentage of dose parameters for each phantom shift for the dose parameter value without the phantom shift in the two modes. Table 2 shows the percentage difference in dose parameters between the two modes. When the 1D phantom shift was <2 voxels, the values of D_98%_, D_95%_, D_50%_, and D_2%_ for VTV and VPNV showed only a small difference between the two modes, of less than approximately 1%. In addition, the differences of D_mean_ and V_40Gy_ for the heart, D_max_ and D1cm^3^ for the spinal cord, and D_mean_ for the thyroid between the two modes were several percent.

**Table 2:** The percentage difference in dose parameters between the NB and DB modes (a) LR, (b) AP, and (c) SI

(a) LR	Shift [voxel]	−3	−2	−1	0	1	2	3
	Shift [mm]	−6.4	−4.3	−2.1	0.0	2.1	4.3	6.4
VTV	D_98%_ [Gy]	−0.2%	0.0%	−0.1%	0.0%	0.0%	−0.1%	−0.7%
	D_95%_ [Gy]	0.0%	0.0%	−0.1%	0.0%	0.0%	−0.1%	−0.4%
	D_50%_ [Gy]	0.1%	0.0%	−0.2%	0.0%	0.0%	−0.1%	−0.1%
	D_2%_ [Gy]	0.2%	0.1%	−0.1%	0.0%	0.0%	−0.1%	−0.2%
VPNV	D_98%_ [Gy]	−0.6%	−0.3%	−0.2%	0.0%	0.1%	−0.1%	−1.0%
	D_95%_ [Gy]	−0.3%	−0.2%	−0.2%	0.0%	0.1%	0.1%	−0.6%
	D_50%_ [Gy]	0.0%	−0.1%	−0.2%	0.0%	0.0%	0.0%	0.0%
	D_2%_ [Gy]	0.2%	0.0%	−0.1%	0.0%	0.0%	−0.1%	0.0%
Heart	D_mean_ [Gy]	0.5%	0.3%	−0.1%	0.0%	−0.2%	−1.0%	−1.6%
	V_40Gy_ [%]	1.0%	0.0%	−0.7%	0.0%	0.3%	−1.1%	−0.2%
Spinal cord	D_max_ [Gy]	−2.9%	−0.6%	0.9%	0.0%	−2.2%	−4.5%	−5.0%
	D1cm^3^ [Gy]	0.0%	0.1%	0.0%	0.0%	−0.1%	−0.1%	−1.1%
Thyroid	D_mean_ [Gy]	0.1%	−0.2%	−0.3%	0.0%	0.1%	−0.5%	−0.2%
(b) AP	Shift [voxel]	−3	−2	−1	0	1	2	3
	Shift [mm]	−6.4	−4.3	−2.1	0.0	2.1	4.3	6.4
VTV	D_98%_ [Gy]	0.0%	−0.1%	−0.1%	0.0%	−0.1%	0.0%	0.0%
	D_95%_ [Gy]	−0.1%	−0.1%	−0.1%	0.0%	0.0%	0.0%	0.0%
	D_50%_ [Gy]	−0.1%	−0.1%	0.0%	0.0%	−0.1%	0.0%	−0.2%
	D_2%_ [Gy]	−0.2%	−0.2%	−0.2%	0.0%	0.0%	0.0%	−0.1%
VPNV	D_98%_ [Gy]	−1.3%	−1.1%	−0.5%	0.0%	0.1%	0.2%	0.6%
	D_95%_ [Gy]	−1.2%	−0.8%	−0.4%	0.0%	0.1%	0.3%	0.1%
	D_50%_ [Gy]	−0.1%	−0.1%	−0.1%	0.0%	−0.1%	0.0%	−0.1%
	D_2%_ [Gy]	0.4%	0.2%	0.0%	0.0%	−0.1%	−0.1%	−0.1%
Heart	D_mean_ [Gy]	−4.7%	−3.4%	−1.5%	0.0%	1.1%	3.4%	4.9%
	V_40Gy_ [%]	−8.6%	−5.8%	−2.7%	0.0%	0.8%	4.9%	7.3%
Spinal cord	D_max_ [Gy]	−5.1%	−4.0%	−2.0%	0.0%	0.8%	−1.4%	−5.0%
	D1cm^3^ [Gy]	−1.9%	−1.5%	−0.9%	0.0%	1.2%	0.7%	0.2%
Thyroid	D_mean_ [Gy]	0.0%	−0.1%	0.1%	0.0%	−1.0%	0.1%	0.1%
(c) SI	Shift [voxel]	−3	−2	−1	0	1	2	3
	Shift [mm]	−6.0	−4.0	−2.0	0.0	2.0	4.0	6.0
VTV	D_98%_ [Gy]	0.0%	0.2%	0.3%	0.0%	0.0%	−0.1%	−0.2%
	D_95%_ [Gy]	0.5%	0.3%	0.2%	0.0%	0.0%	−0.1%	−0.1%
	D_50%_ [Gy]	0.2%	0.1%	0.2%	0.0%	−0.1%	−0.1%	−0.2%
	D_2%_ [Gy]	−0.2%	−0.2%	0.0%	0.0%	0.0%	−0.2%	−0.4%
VPNV	D_98%_ [Gy]	−0.1%	0.0%	0.4%	0.0%	−0.5%	−0.7%	−0.9%
	D_95%_ [Gy]	0.8%	0.7%	0.3%	0.0%	−0.2%	−0.6%	−0.8%
	D_50%_ [Gy]	0.1%	0.0%	0.0%	0.0%	0.0%	0.1%	0.0%
	D_2%_ [Gy]	0.0%	0.0%	0.1%	0.0%	−0.1%	0.0%	−0.1%
Heart	D_mean_ [Gy]	1.6%	1.2%	0.2%	0.0%	−1.2%	−1.8%	−2.3%
	V_40Gy_ [%]	7.3%	5.0%	1.7%	0.0%	−3.6%	−7.4%	−10.3%
Spinal cord	D_max_ [Gy]	−0.6%	0.4%	0.9%	0.0%	−1.4%	−2.1%	−3.8%
	D1cm^3^ [Gy]	0.8%	0.6%	0.8%	0.0%	−0.3%	−0.3%	−0.7%
Thyroid	D_mean_ [Gy]	0.1%	−0.3%	−0.3%	0.0%	0.2%	−0.2%	−0.1%

AP, anterior–posterior; LR, left–right; SI, superior–inferior; VTV, virtual target volume; VPNV, virtual prophylactic node volume; NB, nonblock; DB, directional block.

**Figure 5:**
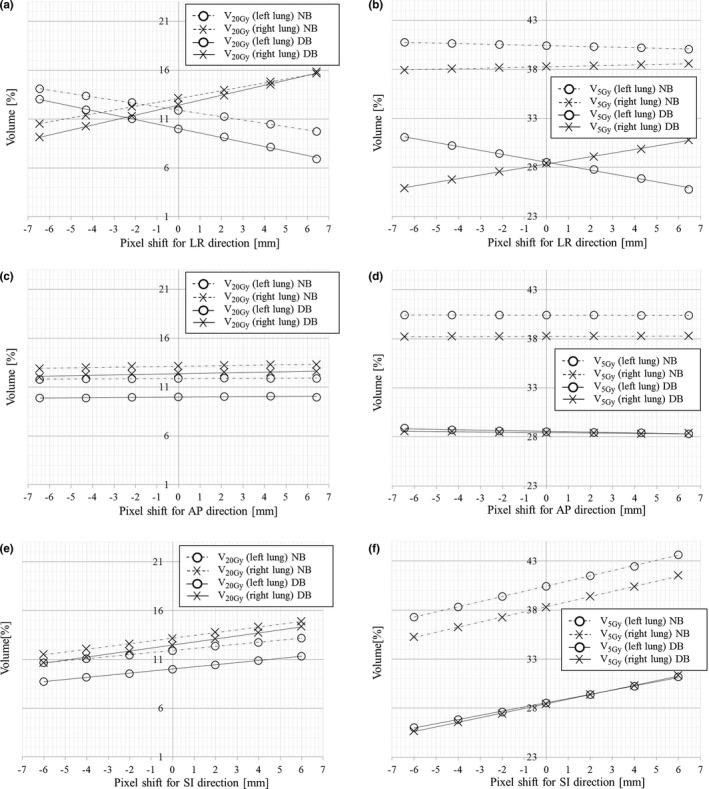
Changes in right and left pulmonary doses (V_20Gy_ and V_5Gy_) related to the shifts in the directional block (DB) and nonblock (NB) modes. (a), (c), (e) V_20Gy_ for shifts in the LR, AP, and SI directions, respectively. (b), (d), (f) V_5Gy_ for shifts in the LR, AP, and SI directions, respectively. AP, anterior–posterior; LR, left–right; SI, superior–inferior.

Figure 5 shows the dose parameters (V_20Gy_ and V_5Gy_) for each of the right and left lungs. The change in V_20Gy_ resulting from the phantom shift in the NB and DB modes was within the treatment planning dose constraints (V_20Gy_ ˂ 25%) [Figs. 5(a), 5(c), and 5(e)]. The other dose parameters (V_10Gy_ and V_15Gy_) were also within the treatment planning dose constraints (data not shown). In addition, the dose parameters in the DB mode were all lower than those in the NB mode. The change rate in V_5Gy_ for the phantom shift in the AP and SI directions in the DB mode was similar to that in the NB mode [Figs. 5(d) and 5(f)], whereas the change rate in the LR direction was different between the NB and DB modes [Fig. 5(b)].

## 4 DISCUSSION

This study quantified the change in doses to the target and OARs resulting from shifts in the phantom when using the DB mode in HT for the treatment plan for cervical esophageal cancer. This is the first report of changes in the dose distribution in the DB mode resulting from phantom shift.

Changes in doses to the clinical target volume (CTV) and OAR resulting from patient movement have been recently reported for the proton^17^, ^18^, ^19^, ^20^ and x‐ray fields.^21^, ^22^, ^23^ Warren et al^21^ reported that the change in the D_98%_ of the CTV was <5% in most cases of volume‐modulated arc radiotherapy for esophageal cancer when the patient shifted ±5 mm in the LR and AP directions and ±7 mm in the SI direction. They set the PTV margin at 5 mm. Additionally, they showed that there were only small changes in D_max_ for the spinal cord, D_mean_ for the heart, and V_20Gy_ for the lungs.^21^ Our study used simple planning models with the RANDO phantom. The results showed that, if a 1D phantom shift in the DB mode was within the same PTV margin as for the NB mode, the change in doses to the target, lungs, heart, and spinal cord was small and within the treatment planning dose constraints. Thus, using the virtual contour shape in this study for a treatment plan in the DB mode for cervical esophageal cancer may keep the dose distribution changes that result from patient movement within a clinically acceptable level. In our previous study, we evaluated the dose reduction to the OARs and dose concentration to the targets using various virtual contour shapes; the virtual contour shape used in the present study was the one that scored the highest in the previous study, which was shown to be the most clinically useful for 20 patients.^10^ This virtual contour shape, a semicircle that follows the shape of the lung at a distance of 8 cm from the tracheal bifurcation, can effectively reduce the dose to OARs and provide safe delivery, even with patient movement, with the same PTV margin as the NB mode.

In our results, the use of the DB mode changed the robustness of the dose distribution around the target, although it did not significantly affect the dose parameters. Lee et al^15^ reported an increase in the dose to the spinal cord by restricting the beamlet that passed through the right hepatic lobe for hepatocellular carcinoma of the left lobe. This was because the dose distribution spread to avoid the virtual contour outline when the DB mode was used.^11^, ^15^ In the present study, the treatment plan using the DB mode increased the beam weight in the AP direction to avoid the bilateral lungs by setting virtual contours. This resulted in a gradual gradient of the medium‐ and low‐dose areas in the AP direction [Fig. 3(b)]. Conversely, the gradient of the medium‐ and low‐dose areas for the LR direction became steep. A gradual dose distribution gradient improves the robustness of the dose with phantom shift, so the dose distribution change resulting from the phantom shift in the DB mode was larger for the LR direction and smaller for the AP direction than those in the NB mode (Fig. 4 and Table 1). Thus, the robustness of a treatment plan using the DB mode would be more affected in the direction parallel to the virtual contour and less affected in the direction perpendicular to the virtual contour. In addition, because the beam weight in the AP direction increases as the bilateral virtual contours becomes closer, the robustness of the dose distribution with respect to the phantom shift in the LR direction would be impaired.

As shown in Fig. 5(b), using the DB mode greatly reduced V_5Gy_ for the lungs compared to using the NB mode, indicating that the DB mode can reduce the risk of radiation pneumonitis. However, V_5Gy_ for the lungs in the DB mode changed greatly with a phantom shift in the LR direction, although that in the NB mode hardly changed. Thus, the DB mode differed from the NB mode in the change of a dose parameter resulting from the phantom shift. Although the dose constraint was satisfied in this study, it is possible that this difference may result in a dose constraint not being satisfied in other treatment cases, depending on the shape and displacement of the virtual contour. This result shows the importance of quantifying the dose uncertainty in the DB mode by confirming the dose distribution change resulting from patient movement.

A limitation of this study was that we confirmed the dose distribution change in the cervical esophageal cancer model by using only 1D phantom shifts. However, real patients move in three dimensions. It is therefore necessary to evaluate whether irradiation is possible with a model of 3D patient movement. In addition, we were not able to perform similar investigations with the TomoDirect™ because we did not have a license of it.

## 5 CONCLUSIONS

For a treatment plan that used the DB mode of HT for cervical esophageal cancer, the change of doses to the target, lungs, heart, and spinal cord would be as small as those of the NB mode if the patient movement was within the same range as the PTV margin of the NB mode. Thus, the virtual contour shape used in this study, a semicircle that followed the shape of the lung at a distance of 8 cm from the tracheal bifurcation, would provide safe delivery when patient movement was within the same PTV margin as the NB mode. However, because the DB mode changed the robustness of the dose distribution around the targets, the dose constraint might not be satisfied depending on the shape and displacement of the virtual contour. Hence, in the DB mode, the robustness of the dose distribution to influence by patient movement should be confirmed with the virtual contour shape used at each facility to ensure the appropriate target and OAR doses.

## CONFLICT OF INTEREST

No conflict of interest.

## Supporting information


**Table S1.** The change in dose parameters by the phantom shift in the nonblock mode [(a) left–right (LR), (b) anterior–posterior (AP), and (c) superior–inferior (SI)].

**Table S2.** The change in dose parameters by the phantom shift in the directional‐block mode [(a) left–right (LR), (b) anterior–posterior (AP), and (c) superior–inferior (SI)].
