# Supplementary material for: Impact of treatment planning using a structure block function on the target and organ doses related to patient movement in cervical esophageal cancer: A phantom study
Source: J Appl Clin Med Phys. 2019 Apr 17;20(5):75–83. doi: 10.1002/acm2.12582 (PMC6523256; doi:10.1002/acm2.12582)
Supplement: Supplementary file 1 — Table S1. The change in dose parameters by the phantom shift in the nonblock mode [(a) left–right (LR), (b) anterior–posterior (AP), and (c) superior–inferior (SI)]. [file ACM2-20-75-s001.docx]

Supplementary file 1 The change in dose parameters by the phantom shift in the non-block mode [(a) LR, (b) AP, and (c) SI]

(a)

| LR | Shift [pixel] | −3 | −2 | −1 | 0 | 1 | 2 | 3 |
| --- | --- | --- | --- | --- | --- | --- | --- | --- |
|  | Shift [mm] | −6.4 | −4.3 | −2.1 | 0.0 | 2.1 | 4.3 | 6.4 |
| VTV | D_98%_ [Gy] | 59.8 | 60.8 | 60.9 | 60.9 | 60.9 | 60.6 | 59.4 |
|  |  | (98.2%) | (99.9%) | (100.1%) | (100.0%) | (100.0%) | (99.6%) | (97.7%) |
|  |  |  |  |  |  |  |  |  |
|  | D_95%_ [Gy] | 60.5 | 60.9 | 61.1 | 61.0 | 61.0 | 60.8 | 60.2 |
|  |  | (99.2%) | (99.9%) | (100.1%) | (100.0%) | (100.0%) | (99.6%) | (98.7%) |
|  |  |  |  |  |  |  |  |  |
|  | D_50%_ [Gy] | 61.5 | 61.6 | 61.7 | 61.6 | 61.6 | 61.5 | 61.4 |
|  |  | (99.9%) | (100.0%) | (100.2%) | (100.0%) | (100.0%) | (99.8%) | (99.7%) |
|  |  |  |  |  |  |  |  |  |
|  | D_2%_ [Gy] | 62.2 | 62.3 | 62.4 | 62.3 | 62.5 | 62.5 | 62.6 |
|  |  | (99.9%) | (99.9%) | (100.1%) | (100.0%) | (100.2%) | (100.3%) | (100.4%) |
|  |  |  |  |  |  |  |  |  |
| VPNV | D_98%_ [Gy] | 48.7 | 49.2 | 49.3 | 49.2 | 49.3 | 49.2 | 48.6 |
|  |  | (99.1%) | (99.9%) | (100.1%) | (100.0%) | (100.1%) | (99.9%) | (98.8%) |
|  |  |  |  |  |  |  |  |  |
|  | D_95%_ [Gy] | 49.4 | 49.7 | 49.8 | 49.8 | 49.8 | 49.7 | 49.3 |
|  |  | (99.2%) | (99.9%) | (100.1%) | (100.0%) | (100.1%) | (99.8%) | (99.0%) |
|  |  |  |  |  |  |  |  |  |
|  | D_50%_ [Gy] | 52.5 | 52.6 | 52.7 | 52.6 | 52.6 | 52.4 | 52.2 |
|  |  | (99.9%) | (100.1%) | (100.2%) | (100.0%) | (100.0%) | (99.7%) | (99.4%) |
|  |  |  |  |  |  |  |  |  |
|  | D2% [Gy] | 62.0 | 61.9 | 62.0 | 61.9 | 62.0 | 62.0 | 62.1 |
|  |  | (100.1%) | (100.0%) | (100.1%) | (100.0%) | (100.1%) | (100.2%) | (100.3%) |
|  |  |  |  |  |  |  |  |  |
| Heart | D_mean_ [Gy] | 6.2 | 6.2 | 6.2 | 6.2 | 6.2 | 6.2 | 6.1 |
|  |  | (99.7%) | (100.2%) | (100.5%) | (100.0%) | (99.7%) | (99.2%) | (97.9%) |
|  |  |  |  |  |  |  |  |  |
|  | V_40Gy_ [%] | 4.8 | 4.9 | 5.0 | 5.0 | 5.0 | 5.0 | 4.8 |
|  |  | (97.0%) | (98.6%) | (100.2%) | (100.0%) | (100.0%) | (100.0%) | (97.0%) |
|  |  |  |  |  |  |  |  |  |
| Spinal cord | D_max_ [Gy] | 41.7 | 41.3 | 40.9 | 40.8 | 41.0 | 40.7 | 40.9 |
|  |  | (102.3%) | (101.4%) | (100.3%) | (100.0%) | (100.5%) | (99.9%) | (100.3%) |
|  |  |  |  |  |  |  |  |  |
|  | D_1cm3_ [Gy] | 38.0 | 37.7 | 37.5 | 37.3 | 37.2 | 37.0 | 37.1 |
|  |  | (102.1%) | (101.2%) | (100.6%) | (100.0%) | (100.0%) | (99.4%) | (99.6%) |
|  |  |  |  |  |  |  |  |  |
| Thyroid | D_mean_ [Gy] | 57.2 | 56.8 | 56.4 | 55.6 | 55.2 | 55.1 | 54.7 |
|  |  | (102.9%) | (102.1%) | (101.4%) | (100.0%) | (99.2%) | (99.0%) | (98.4%) |

(b)

| AP | Shift [pixel] | −3 | −2 | −1 | 0 | 1 | 2 | 3 |
| --- | --- | --- | --- | --- | --- | --- | --- | --- |
|  | Shift [mm] | −6.4 | −4.3 | −2.1 | 0.0 | 2.1 | 4.3 | 6.4 |
| VTV | D_98%_ [Gy] | 60.1 | 61.2 | 61.1 | 60.9 | 60.6 | 59.8 | 57.6 |
|  |  | (98.8%) | (100.5%) | (100.4%) | (100.0%) | (99.5%) | (98.3%) | (94.6%) |
|  |  |  |  |  |  |  |  |  |
|  | D_95%_ [Gy] | 61.0 | 61.4 | 61.2 | 61.0 | 60.7 | 60.2 | 58.6 |
|  |  | (99.9%) | (100.6%) | (100.4%) | (100.0%) | (99.5%) | (98.6%) | (96.1%) |
|  |  |  |  |  |  |  |  |  |
|  | D_50%_ [Gy] | 62.3 | 62.2 | 61.9 | 61.6 | 61.3 | 61.0 | 60.7 |
|  |  | (101.2%) | (101.0%) | (100.5%) | (100.0%) | (99.5%) | (99.1%) | (98.6%) |
|  |  |  |  |  |  |  |  |  |
|  | D_2%_ [Gy] | 63.3 | 63.0 | 62.7 | 62.3 | 62.1 | 62.0 | 61.9 |
|  |  | (101.6%) | (101.1%) | (100.6%) | (100.0%) | (99.7%) | (99.5%) | (99.2%) |
|  |  |  |  |  |  |  |  |  |
| VPNV | D_98%_ [Gy] | 48.5 | 49.2 | 49.3 | 49.2 | 49.1 | 48.8 | 47.8 |
|  |  | (98.7%) | (100.0%) | (100.1%) | (100.0%) | (99.9%) | (99.2%) | (97.2%) |
|  |  |  |  |  |  |  |  |  |
|  | D_95%_ [Gy] | 49.4 | 49.8 | 49.9 | 49.8 | 49.6 | 49.3 | 48.6 |
|  |  | (99.2%) | (100.1%) | (100.3%) | (100.0%) | (99.7%) | (99.0%) | (97.7%) |
|  |  |  |  |  |  |  |  |  |
|  | D_50%_ [Gy] | 52.7 | 52.8 | 52.7 | 52.6 | 52.4 | 52.1 | 51.7 |
|  |  | (100.3%) | (100.4%) | (100.3%) | (100.0%) | (99.7%) | (99.1%) | (98.3%) |
|  |  |  |  |  |  |  |  |  |
|  | D_2%_ [Gy] | 62.6 | 62.4 | 62.2 | 61.9 | 61.7 | 61.3 | 61.0 |
|  |  | (101.1%) | (100.8%) | (100.4%) | (100.0%) | (99.6%) | (99.1%) | (98.6%) |
|  |  |  |  |  |  |  |  |  |
| Heart | D_mean_ [Gy] | 6.8 | 6.6 | 6.4 | 6.2 | 6.0 | 5.8 | 5.5 |
|  |  | (109.8%) | (106.9%) | (103.4%) | (100.0%) | (96.8%) | (92.6%) | (88.9%) |
|  |  |  |  |  |  |  |  |  |
|  | V_40Gy_ [%] | 6.6 | 6.1 | 5.5 | 5.0 | 4.5 | 3.8 | 3.3 |
|  |  | (132.5%) | (122.2%) | (110.9%) | (100.0%) | (89.9%) | (77.2%) | (65.9%) |
|  |  |  |  |  |  |  |  |  |
| Spinal cord | D_max_ [Gy] | 37.1 | 38.3 | 39.6 | 40.8 | 42.3 | 44.9 | 48.3 |
|  |  | (91.1%) | (94.1%) | (97.1%) | (100.0%) | (103.9%) | (110.3%) | (118.6%) |
|  |  |  |  |  |  |  |  |  |
|  | D_1cm3_ [Gy] | 34.5 | 35.3 | 36.2 | 37.3 | 38.5 | 40.5 | 43.0 |
|  |  | (92.5%) | (94.7%) | (97.2%) | (100.0%) | (103.3%) | (108.7%) | (115.4%) |
|  |  |  |  |  |  |  |  |  |
| Thyroid | D_mean_ [Gy] | 61.1 | 59.9 | 57.9 | 55.6 | 53.2 | 49.9 | 47.2 |
|  |  | (109.8%) | (107.7%) | (104.2%) | (100.0%) | (95.6%) | (89.7%) | (84.8%) |

(c)

| SI | Shift [pixel] | −3 | −2 | −1 | 0 | 1 | 2 | 3 |
| --- | --- | --- | --- | --- | --- | --- | --- | --- |
|  | Shift [mm] | −6.0 | −4.0 | −2.0 | 0.0 | 2.0 | 4.0 | 6.0 |
| VTV | D_98%_ [Gy] | 59.8 | 60.5 | 60.7 | 60.9 | 60.5 | 60.0 | 59.2 |
|  |  | (98.2%) | (99.5%) | (99.8%) | (100.0%) | (99.3%) | (98.5%) | (97.2%) |
|  |  |  |  |  |  |  |  |  |
|  | D_95%_ [Gy] | 60.4 | 60.8 | 61.0 | 61.0 | 60.7 | 60.1 | 59.4 |
|  |  | (99.0%) | (99.6%) | (99.9%) | (100.0%) | (99.4%) | (98.6%) | (97.3%) |
|  |  |  |  |  |  |  |  |  |
|  | D_50%_ [Gy] | 61.9 | 61.9 | 61.7 | 61.6 | 61.4 | 61.3 | 61.0 |
|  |  | (100.6%) | (100.5%) | (100.2%) | (100.0%) | (99.8%) | (99.6%) | (99.2%) |
|  |  |  |  |  |  |  |  |  |
|  | D_2%_ [Gy] | 64.0 | 63.4 | 62.7 | 62.3 | 62.3 | 62.5 | 62.6 |
|  |  | (102.6%) | (101.7%) | (100.6%) | (100.0%) | (100.0%) | (100.3%) | (100.4%) |
|  |  |  |  |  |  |  |  |  |
| VPNV | D_98%_ [Gy] | 48.3 | 49.0 | 49.2 | 49.2 | 49.1 | 48.6 | 48.0 |
|  |  | (98.2%) | (99.6%) | (99.9%) | (100.0%) | (99.7%) | (98.8%) | (97.5%) |
|  |  |  |  |  |  |  |  |  |
|  | D_95%_ [Gy] | 49.1 | 49.5 | 49.8 | 49.8 | 49.6 | 49.2 | 48.7 |
|  |  | (98.6%) | (99.5%) | (100.0%) | (100.0%) | (99.6%) | (98.9%) | (97.8%) |
|  |  |  |  |  |  |  |  |  |
|  | D_50%_ [Gy] | 52.6 | 52.6 | 52.6 | 52.6 | 52.5 | 52.4 | 52.2 |
|  |  | (100.1%) | (100.1%) | (100.0%) | (100.0%) | (99.8%) | (99.6%) | (99.3%) |
|  |  |  |  |  |  |  |  |  |
|  | D_2%_ [Gy] | 63.0 | 62.6 | 62.1 | 61.9 | 61.9 | 62.0 | 62.0 |
|  |  | (101.7%) | (101.1%) | (100.4%) | (100.0%) | (100.0%) | (100.1%) | (100.2%) |
|  |  |  |  |  |  |  |  |  |
| Heart | D_mean_ [Gy] | 5.1 | 5.4 | 5.8 | 6.2 | 6.7 | 7.1 | 7.5 |
|  |  | (81.6%) | (87.4%) | (93.6%) | (100.0%) | (107.2%) | (113.8%) | (121.1%) |
|  |  |  |  |  |  |  |  |  |
|  | V_40Gy_ [%] | 3.5 | 3.9 | 4.4 | 5.0 | 5.6 | 6.1 | 6.7 |
|  |  | (70.4%) | (79.4%) | (89.5%) | (100.0%) | (111.9%) | (123.4%) | (134.5%) |
|  |  |  |  |  |  |  |  |  |
| Spinal cord | D_max_ [Gy] | 43.6 | 42.5 | 41.5 | 40.8 | 40.2 | 39.6 | 38.9 |
|  |  | (107.0%) | (104.2%) | (101.7%) | (100.0%) | (98.7%) | (97.1%) | (95.5%) |
|  |  |  |  |  |  |  |  |  |
|  | D_1cm3_ [Gy] | 39.4 | 38.7 | 37.9 | 37.3 | 36.6 | 36.1 | 35.4 |
|  |  | (105.8%) | (103.9%) | (101.7%) | (100.0%) | (98.3%) | (96.8%) | (95.1%) |
|  |  |  |  |  |  |  |  |  |
| Thyroid | D_mean_ [Gy] | 56.1 | 56.0 | 55.9 | 55.6 | 55.4 | 55.4 | 55.1 |
|  |  | (100.9%) | (100.7%) | (100.4%) | (100.0%) | (99.6%) | (99.7%) | (99.1%) |

AP, anterior-posterior; LR, left-right; SI, superior-inferior; VTV, virtual target volume; VPNV, virtual prophylactic node volume
