# Supplementary material for: Impact of treatment planning using a structure block function on the target and organ doses related to patient movement in cervical esophageal cancer: A phantom study
Source: J Appl Clin Med Phys. 2019 Apr 17;20(5):75–83. doi: 10.1002/acm2.12582 (PMC6523256; doi:10.1002/acm2.12582)
Supplement: Supplementary file 2 — Table S2. The change in dose parameters by the phantom shift in the directional‐block mode [(a) left–right (LR), (b) anterior–posterior (AP), and (c) superior–inferior (SI)]. [file ACM2-20-75-s002.docx]

Supplementary file 2 The change in dose parameters by the phantom shift in the directional-block mode [(a) LR, (b) AP, and (c) SI]

(a)

| LR | Shift [pixel] | −3 | −2 | −1 | 0 | 1 | 2 | 3 |
| --- | --- | --- | --- | --- | --- | --- | --- | --- |
|  | Shift [mm] | −6.4 | −4.3 | −2.1 | 0.0 | 2.1 | 4.3 | 6.4 |
| VTV | D_98%_ [Gy] | 59.7 | 60.9 | 60.9 | 60.9 | 60.9 | 60.6 | 59.1 |
|  |  | (98.0%) | (99.9%) | (100.0%) | (100.0%) | (100.0%) | (99.4%) | (97.0%) |
|  |  |  |  |  |  |  |  |  |
|  | D_95%_ [Gy] | 60.5 | 61.0 | 61.1 | 61.1 | 61.1 | 60.8 | 60.0 |
|  |  | (99.1%) | (99.9%) | (100.0%) | (100.0%) | (100.0%) | (99.5%) | (98.3%) |
|  |  |  |  |  |  |  |  |  |
|  | D_50%_ [Gy] | 61.7 | 61.7 | 61.7 | 61.7 | 61.7 | 61.5 | 61.4 |
|  |  | (100.0%) | (100.0%) | (100.0%) | (100.0%) | (100.0%) | (99.8%) | (99.6%) |
|  |  |  |  |  |  |  |  |  |
|  | D_2%_ [Gy] | 62.5 | 62.5 | 62.5 | 62.5 | 62.6 | 62.6 | 62.6 |
|  |  | (100.1%) | (100.0%) | (100.0%) | (100.0%) | (100.2%) | (100.2%) | (100.3%) |
|  |  |  |  |  |  |  |  |  |
| VPNV | D_98%_ [Gy] | 48.0 | 48.6 | 48.7 | 48.8 | 48.9 | 48.7 | 47.7 |
|  |  | (98.5%) | (99.6%) | (99.9%) | (100.0%) | (100.2%) | (99.9%) | (97.8%) |
|  |  |  |  |  |  |  |  |  |
|  | D_95%_ [Gy] | 49.0 | 49.4 | 49.5 | 49.5 | 49.6 | 49.5 | 48.7 |
|  |  | (98.9%) | (99.7%) | (99.9%) | (100.0%) | (100.2%) | (99.9%) | (98.4%) |
|  |  |  |  |  |  |  |  |  |
|  | D_50%_ [Gy] | 52.4 | 52.4 | 52.5 | 52.4 | 52.4 | 52.3 | 52.1 |
|  |  | (99.9%) | (100.0%) | (100.1%) | (100.0%) | (100.0%) | (99.7%) | (99.4%) |
|  |  |  |  |  |  |  |  |  |
|  | D2% [Gy] | 62.1 | 62.0 | 61.9 | 61.9 | 62.0 | 62.0 | 62.1 |
|  |  | (100.3%) | (100.0%) | (100.0%) | (100.0%) | (100.1%) | (100.1%) | (100.3%) |
|  |  |  |  |  |  |  |  |  |
| Heart | D_mean_ [Gy] | 7.1 | 7.1 | 7.1 | 7.1 | 7.0 | 6.9 | 6.8 |
|  |  | (100.1%) | (100.4%) | (100.4%) | (100.0%) | (99.4%) | (98.2%) | (96.3%) |
|  |  |  |  |  |  |  |  |  |
|  | V_40Gy_ [%] | 6.4 | 6.4 | 6.5 | 6.5 | 6.5 | 6.4 | 6.3 |
|  |  | (98.0%) | (98.6%) | (99.5%) | (100.0%) | (100.3%) | (98.9%) | (96.8%) |
|  |  |  |  |  |  |  |  |  |
| Spinal cord | D_max_ [Gy] | 42.1 | 42.7 | 42.9 | 42.4 | 41.7 | 40.5 | 40.4 |
|  |  | (99.4%) | (100.8%) | (101.2%) | (100.0%) | (98.3%) | (95.5%) | (95.3%) |
|  |  |  |  |  |  |  |  |  |
|  | D_1cm3_ [Gy] | 38.9 | 38.6 | 38.3 | 38.1 | 38.0 | 37.8 | 37.5 |
|  |  | (102.0%) | (101.3%) | (100.6%) | (100.0%) | (99.8%) | (99.2%) | (98.5%) |
|  |  |  |  |  |  |  |  |  |
| Thyroid | D_mean_ [Gy] | 57.3 | 56.8 | 56.3 | 55.7 | 55.3 | 54.9 | 54.7 |
|  |  | (103.0%) | (102.0%) | (101.0%) | (100.0%) | (99.3%) | (98.5%) | (98.2%) |

(b)

| AP | Shift [pixel] | −3 | −2 | −1 | 0 | 1 | 2 | 3 |
| --- | --- | --- | --- | --- | --- | --- | --- | --- |
|  | Shift [mm] | −6.4 | −4.3 | −2.1 | 0.0 | 2.1 | 4.3 | 6.4 |
| VTV | D_98%_ [Gy] | 60.2 | 61.2 | 61.1 | 60.9 | 60.6 | 59.9 | 57.7 |
|  |  | (98.8%) | (100.4%) | (100.3%) | (100.0%) | (99.4%) | (98.3%) | (94.6%) |
|  |  |  |  |  |  |  |  |  |
|  | D_95%_ [Gy] | 60.9 | 61.4 | 61.3 | 61.1 | 60.7 | 60.2 | 58.7 |
|  |  | (99.8%) | (100.5%) | (100.3%) | (100.0%) | (99.4%) | (98.6%) | (96.1%) |
|  |  |  |  |  |  |  |  |  |
|  | D_50%_ [Gy] | 62.4 | 62.2 | 62.0 | 61.7 | 61.3 | 61.1 | 60.7 |
|  |  | (101.2%) | (100.9%) | (100.5%) | (100.0%) | (99.5%) | (99.0%) | (98.4%) |
|  |  |  |  |  |  |  |  |  |
|  | D_2%_ [Gy] | 63.4 | 63.1 | 62.7 | 62.5 | 62.3 | 62.2 | 62.0 |
|  |  | (101.4%) | (100.9%) | (100.4%) | (100.0%) | (99.7%) | (99.5%) | (99.2%) |
|  |  |  |  |  |  |  |  |  |
| VPNV | D_98%_ [Gy] | 47.5 | 48.3 | 48.6 | 48.8 | 48.8 | 48.5 | 47.7 |
|  |  | (97.4%) | (99.0%) | (99.7%) | (100.0%) | (100.0%) | (99.3%) | (97.8%) |
|  |  |  |  |  |  |  |  |  |
|  | D_95%_ [Gy] | 48.6 | 49.2 | 49.4 | 49.5 | 49.4 | 49.1 | 48.5 |
|  |  | (98.1%) | (99.3%) | (99.8%) | (100.0%) | (99.8%) | (99.2%) | (97.8%) |
|  |  |  |  |  |  |  |  |  |
|  | D_50%_ [Gy] | 52.5 | 52.6 | 52.5 | 52.4 | 52.2 | 51.9 | 51.5 |
|  |  | (100.2%) | (100.3%) | (100.2%) | (100.0%) | (99.6%) | (99.1%) | (98.2%) |
|  |  |  |  |  |  |  |  |  |
|  | D_2%_ [Gy] | 62.8 | 62.5 | 62.2 | 61.9 | 61.6 | 61.3 | 61.0 |
|  |  | (101.5%) | (101.0%) | (100.5%) | (100.0%) | (99.5%) | (99.0%) | (98.4%) |
|  |  |  |  |  |  |  |  |  |
| Heart | D_mean_ [Gy] | 7.4 | 7.3 | 7.2 | 7.1 | 6.9 | 6.8 | 6.6 |
|  |  | (105.1%) | (103.5%) | (101.8%) | (100.0%) | (97.9%) | (96.0%) | (93.8%) |
|  |  |  |  |  |  |  |  |  |
|  | V_40Gy_ [%] | 8.0 | 7.6 | 7.0 | 6.5 | 5.9 | 5.3 | 4.8 |
|  |  | (123.9%) | (116.3%) | (108.2%) | (100.0%) | (90.8%) | (82.1%) | (73.2%) |
|  |  |  |  |  |  |  |  |  |
| Spinal cord | D_max_ [Gy] | 36.5 | 38.2 | 40.3 | 42.4 | 44.4 | 46.2 | 48.1 |
|  |  | (86.0%) | (90.1%) | (95.0%) | (100.0%) | (104.7%) | (108.9%) | (113.6%) |
|  |  |  |  |  |  |  |  |  |
|  | D_1cm3_ [Gy] | 34.5 | 35.5 | 36.7 | 38.1 | 39.8 | 41.7 | 44.0 |
|  |  | (90.6%) | (93.2%) | (96.4%) | (100.0%) | (104.5%) | (109.4%) | (115.6%) |
|  |  |  |  |  |  |  |  |  |
| Thyroid | D_mean_ [Gy] | 61.1 | 59.9 | 58.1 | 55.7 | 52.7 | 50.0 | 47.3 |
|  |  | (109.8%) | (107.6%) | (104.2%) | (100.0%) | (94.6%) | (89.8%) | (84.9%) |

(c)

| SI | Shift [pixel] | −3 | −2 | −1 | 0 | 1 | 2 | 3 |
| --- | --- | --- | --- | --- | --- | --- | --- | --- |
|  | Shift [mm] | −6.0 | −4.0 | −2.0 | 0.0 | 2.0 | 4.0 | 6.0 |
| VTV | D_98%_ [Gy] | 59.9 | 60.8 | 61.0 | 60.9 | 60.5 | 60.0 | 59.1 |
|  |  | (98.2%) | (99.7%) | (100.1%) | (100.0%) | (99.3%) | (98.4%) | (97.0%) |
|  |  |  |  |  |  |  |  |  |
|  | D_95%_ [Gy] | 60.8 | 61.0 | 61.2 | 61.1 | 60.7 | 60.2 | 59.3 |
|  |  | (99.5%) | (99.9%) | (100.1%) | (100.0%) | (99.4%) | (98.5%) | (97.2%) |
|  |  |  |  |  |  |  |  |  |
|  | D_50%_ [Gy] | 62.1 | 62.0 | 61.9 | 61.7 | 61.5 | 61.4 | 61.0 |
|  |  | (100.8%) | (100.6%) | (100.4%) | (100.0%) | (99.7%) | (99.5%) | (99.0%) |
|  |  |  |  |  |  |  |  |  |
|  | D_2%_ [Gy] | 64.0 | 63.4 | 62.9 | 62.5 | 62.4 | 62.5 | 62.5 |
|  |  | (102.5%) | (101.5%) | (100.6%) | (100.0%) | (100.0%) | (100.0%) | (100.0%) |
|  |  |  |  |  |  |  |  |  |
| VPNV | D_98%_ [Gy] | 47.9 | 48.6 | 48.9 | 48.8 | 48.4 | 47.9 | 47.1 |
|  |  | (98.1%) | (99.6%) | (100.3%) | (100.0%) | (99.2%) | (98.2%) | (96.6%) |
|  |  |  |  |  |  |  |  |  |
|  | D_95%_ [Gy] | 49.3 | 49.6 | 49.7 | 49.5 | 49.2 | 48.7 | 48.0 |
|  |  | (99.5%) | (100.2%) | (100.4%) | (100.0%) | (99.4%) | (98.3%) | (97.0%) |
|  |  |  |  |  |  |  |  |  |
|  | D_50%_ [Gy] | 52.5 | 52.5 | 52.4 | 52.4 | 52.4 | 52.3 | 52.1 |
|  |  | (100.2%) | (100.1%) | (100.0%) | (100.0%) | (99.9%) | (99.7%) | (99.3%) |
|  |  |  |  |  |  |  |  |  |
|  | D2_%_ [Gy] | 63.0 | 62.6 | 62.2 | 61.9 | 61.9 | 62.0 | 62.0 |
|  |  | (101.7%) | (101.0%) | (100.5%) | (100.0%) | (99.9%) | (100.1%) | (100.1%) |
|  |  |  |  |  |  |  |  |  |
| Heart | D_mean_ [Gy] | 5.9 | 6.3 | 6.6 | 7.1 | 7.5 | 7.9 | 8.4 |
|  |  | (83.3%) | (88.7%) | (93.8%) | (100.0%) | (106.1%) | (112.0%) | (118.8%) |
|  |  |  |  |  |  |  |  |  |
|  | V_40Gy_ [%] | 5.0 | 5.5 | 5.9 | 6.5 | 7.0 | 7.5 | 8.1 |
|  |  | (77.7%) | (84.4%) | (91.2%) | (100.0%) | (108.3%) | (116.0%) | (124.2%) |
|  |  |  |  |  |  |  |  |  |
| Spinal cord | D_max_ [Gy] | 45.1 | 44.3 | 43.5 | 42.4 | 41.2 | 40.3 | 38.9 |
|  |  | (106.3%) | (104.6%) | (102.6%) | (100.0%) | (97.3%) | (95.1%) | (91.7%) |
|  |  |  |  |  |  |  |  |  |
|  | D_1cm3_ [Gy] | 40.6 | 39.8 | 39.0 | 38.1 | 37.4 | 36.8 | 36.0 |
|  |  | (106.6%) | (104.4%) | (102.4%) | (100.0%) | (98.1%) | (96.5%) | (94.4%) |
|  |  |  |  |  |  |  |  |  |
| Thyroid | D_mean_ [Gy] | 56.2 | 56.0 | 55.8 | 55.7 | 55.6 | 55.4 | 55.1 |
|  |  | (101.0%) | (100.5%) | (100.2%) | (100.0%) | (99.8%) | (99.5%) | (99.0%) |

AP, anterior-posterior; LR, left-right; SI, superior-inferior; VTV, virtual target volume; VPNV, virtual prophylactic node volume
